# A life-cycle assessment framework for quantifying the carbon footprint of rural households based on survey data

**DOI:** 10.1016/j.mex.2021.101411

**Published:** 2021-06-09

**Authors:** Yechennan Peng, Liang Emlyn Yang, Jürgen Scheffran

**Affiliations:** a Institute of Geography, Center for Earth System Research and Sustainability (CEN), University of Hamburg, Germany; b School of Integrated Climate System Sciences, University of Hamburg, Germany; c Institute of Pre- and Protohistoric Archaeology, Kiel University, Germany

**Keywords:** Life-cycle approach, Carbon emission, Carbon sequestration, Household livelihood, Livelihood transition, Rural areas

## Abstract

This paper presents the development of a life-cycle assessment (LCA) framework for quantifying the carbon footprint of individual households based on detailed household survey data. According to household consumption and production patterns, the comprehensive life-cycle assessment framework is designed with clarified life-cycle boundaries. The framework covers eight types of specific living activities of rural households generated from a large-scale household survey in the Three Gorges Reservoir area in China. Both carbon emissions and carbon sequestrations of households’ consumption and production activities can be assessed, and eventually the integrative carbon footprint is evaluated. The LCA framework enables comprehensive and relatively precise assessment of carbon footprint at a household level and thus adds value to studies in the fields of carbon emission reduction, carbon neutrality and climate policies.

## Highlights

• We developed an improved Life-Cycle Assessment framework for carbon footprint evaluation.
• The LCA frameworks can identify the life-cycle boundaries of household activities.
• The improved LCA framework helps identify specific producing and consuming activities of rural households.
• The LCA framework enables a comprehensive and relatively precise assessment of carbon footprint at household level.

## Graphical abstract

**Figure:**
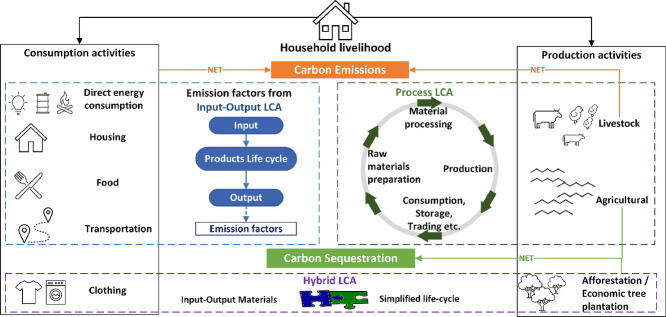

Specifications table

**Table:** 

Subject Area:	Environmental Science
More specific subject area:	carbon footprint, carbon emission, rural livelihood
Method name:	Life cycle assessment framework of households’ carbon footprint
Name and reference of original method:	Life cycle assessment M. Z. Hauschild, R. K. Rosenbaum, and S. I. Olsen, Life Cycle Assessment Theory and Practice. Springer, 2018.
Resource availability:	Excel spreadsheet

## 1 Background

The approach of life-cycle assessment (LCA) facilitates a systematical view in environmental evaluation of a product from raw material extraction, manufacturing, and utilization to ultimate disposal [1], [2], [3]. Since the 2000s, the LCA had been frequently applied to evaluate various environmental impacts of human activities [4]. The scope of LCA was broadened from the environmental influences of product-related research (product level) to process-related research (process level) or activity-related research. Specifically, the applied methods and models to evaluate these levels include process-LCA, environmental input-output LCA (EIO-LCA) and hybrid LCA.

Overall, LCA is widely applied to evaluate the environmental impacts of product, process, or activity. First, an individual product's environmental impact could be assessed within a LCA framework, such as the different environmental impacts of various building materials [5]. Second, LCA can also assess an aggregated environmental impact of a process with a group of materials. A good example is that Petrovic applied the process-LCA to identify the global warming potential of building a single-family house in Sweden [6]. In addition, the impacts of human activities that involve several different processes could also be quantified with a LCA approach. For instance, Ivanova evaluates the environmental impact of household consumption activities in terms of housing, clothing, mobility and so on [7].

Despite the broad application of LCA in specific individual production processes or aggregated activities, no explicit framework has been designed to assess the carbon footprint of overall household activities based on survey data. The existing research has either focused on specific household activities [8,9], applying statistical data in an input and output approach [10], or focusing on more macro-level social networks [11]. An overall LCA framework is thus required to quantify the carbon footprint based on both production and consumption activities at the household level.

This study developed an improved LCA framework for quantifying the carbon footprint of rural households. The framework aims to assess carbon emissions and carbon sequestrations of households' living and production activities and eventually evaluate their integrative carbon footprint. The present paper introduces especially the methodology framework, the assessment process and the data requirements. A separate co-submission paper covers the application of the method in the Three Gorges Reservoir (TGR) area in China, where details of the data collection, data analysis, research area, results analysis, and relevant policy indications were introduced.

## 2 LCA framework for quantifying carbon footprint

**Fig. 1:**
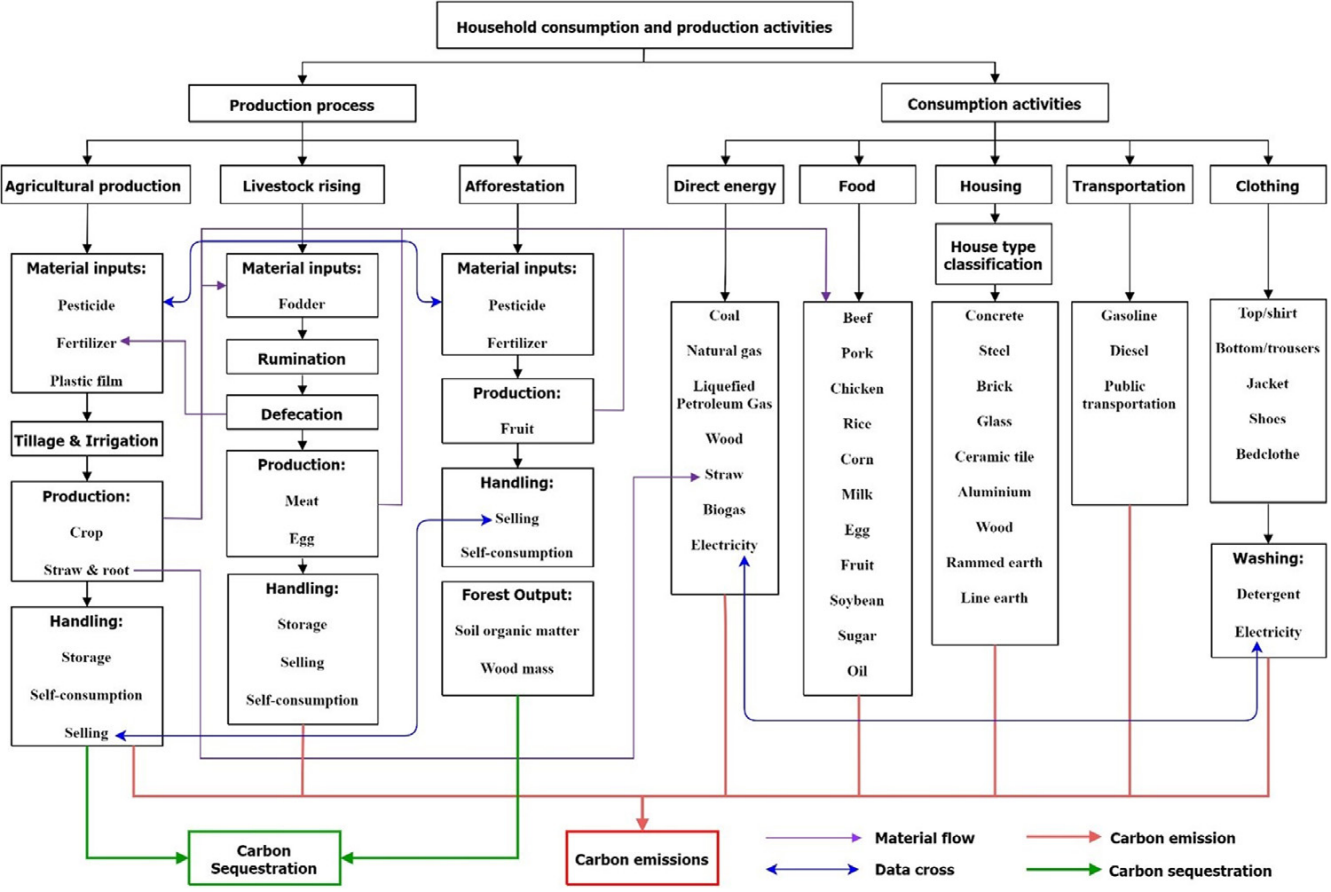
The framework for calculating carbon footprint, covering both carbon emissions and carbon sequestrations at the household level.

This study designed a framework for calculating the carbon footprint based on households' consumption and production patterns (Fig. 1). In the framework, the carbon footprint is calculated using three types of life cycle assessment (LCA) [12]:

• Process LCA for agriculture and livestock related processes.
• Input–output LCA for consumptions of energy, food, housing and transportation.
• Hybrid LCA for afforestation processes in production and the clothing in household consumptions.

The consumption activities of a rural household cover direct energy consumption (e.g. fuel, electricity) and other consumption of food, housing, transport, and clothes. The production and processing stages of these goods all involve energy consumption and associated carbon emissions. The household production activities include agriculture, livestock rising and economic afforestation according to a field survey with the households. On the one hand, carbon-capturing processes occurred through biomass growth and soil carbon sink in agriculture and forestry. On the other hand, these production activities also emit carbon through the material inputs, including fertilizers, pesticides and plastic films during the crop planting process. Both emission and sequestration processes are assessed separately in the present LCA framework (Fig. 1) to evaluate a net carbon footprint.

The household carbon footprint is assessed in the scope of living consumptions, household production, and the production chain of the consumed products. Fig. 1 shows that some materials in the household production and consumption sectors are overlapping or complementary to each other, as connected by the purple lines. The blue lines link the sectors that have input-output relations. Carbon footprint calculations of these sectors are therefore especially treated to avoid duplications. By carefully considering these complex material flows and data cross issues, our improved LCA approach could identify the repeating or missing items and thus ensure the accuracy of the overall assessment. More details are further presented in Section 3.

A specific application of the framework was carried out using households survey data in the Three Gorges Reservoir (TGR) area in western China, and the results are presented in a separate co-submission paper.

## 3 Assessment of carbon footprint

The overall carbon footprint of households is calculated using Eq. 1, and specific assessments of each category are presented in the following Sections 3.1-3.5:

(1) $$\text{Carbon}\;\text{flu}x_{i}=\left(\sum^{n}\text{Carbon}\;\text{emission}s_{\text{in}}+\sum^{m}\text{Carbon}\;\text{sequestration}s_{\text{im}}\right)$$

– $\text{Carbon}\;\text{flu}x_{i}$ represents the sum of per capita carbon emission and carbon sequestration of the household i.
– $\text{Carbon}\;\text{emissio}n_{\text{in}}$ is the household i annual carbon emissions in category n.
– $\text{Carbon}\;\text{sequestratio}n_{\text{im}}$ is the household annual carbon sequestration of in category m.

### 3.1 Carbon emissions of direct energy consumption

*Carbon emissions of direct energy* are mainly generated from the consumption of fossil fuel, biofuel and electricity. The annual carbon emission of household i from the direct energy thus depends on the fuel type d, the amount of fuel consumption ($\text{Fue}l_{\text{id}}$) and the relevant carbon emission factors ($\text{Emission}\;\text{facto}r_{d}$) by equation 2:

(2) $$\text{Carbon}\;\text{emissio}n_{\text{id}}=\sum \left(\text{Fue}l_{\text{id}}*\;\text{Emission}\;\text{facto}r_{d}\right)$$

– $\text{Carbon}\;\text{emissio}n_{\text{id}}$ represents the carbon emission from direct fuel consumption of the household i.

The carbon emission factors of each fossil fuel are defined by Eq. 3, which derives from existing studies, especially for cases in China [13,14]:

(3) $$\text{Emission}\;\text{facto}r_{d}=OX_{d}*\left(C_{o,d}*\frac{12}{44}+C_{h,d}*\frac{12}{16}\right)*H_{d}/10^{-9}$$

Here, the default $CO_{2}\;$emission factor ($C_{o,d}$) and $CH_{4}$ emission factor ($C_{h,d}$) are adopted from the IPCC AR 5 [15] and the net calorific basis ($H_{d}$) of the GHG Protocol [16]. At the same time, we assume the oxygenation efficiency ($OX_{d}$) is at the perfect level of 100%.

The carbon emission factors of biofuels and electricity are determined by Eq. 4, including the emission factor of standard coal ($\text{Emission}\;\text{facto}r_{\text{SC}}$) and the standard coal coefficient ($\text{SC}\;\text{coefficient}{\;}_{d}$) of each biofuel or electricity.

(4) $$\text{Emission}\;\text{facto}r_{d}=\text{Emission}\;\text{facto}r_{\text{SC}}*\text{SC}\;\text{coefficien}t_{d}$$

### 3.2 Carbon emissions in living consumptions

Carbon emissions also occurred in indirect carbon consumption of the households, including consumed clothing, food, housing and transportation. The consumptions could be separated into short-lived consumer products and durable consumer products. The annual carbon emissions of the short-lived consumer products, including food products and vehicle fuels, are simply calculated by the function of the product (type f) emission factors and the amount of consumed products (Eq. 5). For the durable consumer products, housing and clothing, the product lifetime should be considered in the annual carbon emissions function, as illustrated in equation 6:

(5) $$\text{Carbon}\;\text{emissio}n_{\text{if}}=\sum \left(\text{Emission}\;\text{facto}r_{f}\;*\text{Consumed}\;\text{materia}l_{\text{if}}\right)$$

(6) $$\text{Carbon}\;\text{emissio}n_{\text{ij}}=\sum \left(\text{Emission}\;\text{facto}r_{j}\;*\text{Consumed}\;\text{materia}l_{\text{ij}}\right)/\text{Lifetim}e_{j}$$

– $\text{Carbon}\;\text{emissio}n_{\text{if}}$ represents the carbon emission from short-lived consumer products of the household i.
– $\text{Carbon}\;\text{emissio}n_{\text{ij}}$ represents the carbon emission from durable consumer products of the household i.

The carbon emission factors of various indirect consumption are adopted from literature references. Specifically, carbon emissions from both self-produced food and purchased food are all considered in the calculation by different types and with different emission factors. In addition, the consumption data in the transportation category are mostly the spending of fuels or public transportation, which should be first transformed into the amount of fuel according to the average price of the fuel or the ticket price of the public transportation in the same year. Afterwards, the carbon emission of transportation can be calculated by the amount of the consumed fuels and its relevant emission factors.

The calculation of carbon emissions from clothing follows the carbon emission functions of durable consumer products in response to the long lifetime of clothes (2–10 years varied by different clothes). The detergents and electricity consumed in the washing processes are also considered as short-lived products. Besides, electricity consumption is not repetitively included in the category of clothing consumption because the overall household electricity consumption has been included in the direct energy consumption category. The other durable consumer products and housing were also approximately calculated according to different housing structures [17]. In principle, six types of building structures are identified in this current research: reinforced brick-concrete structure, brick structure, brick wood structure, rammed earth house, conventional wood structure and straw building. Since house owners in China have legal ownership of house properties for only 70 years, the lifetime of all residential buildings is set for 70 years.

### 3.3 Carbon footprint in agricultural activities

**Fig. 2:**
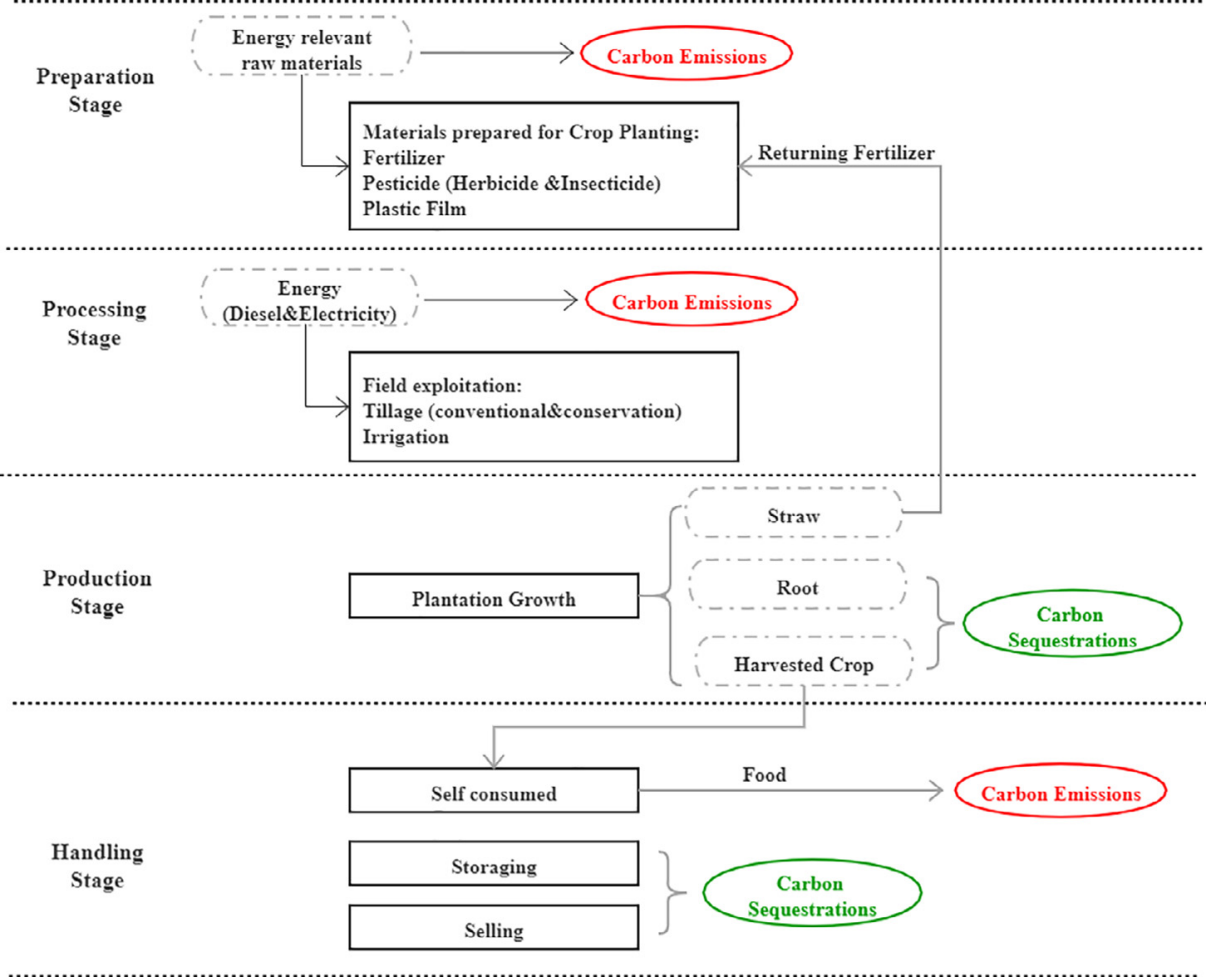
Life Cycle process of agricultural activities.

Carbon footprint processes were assessed in many agricultural activities (agriculture, afforestation). Though with large amount of labor migrations, crop productions have been keeping increasing in West China [18]. Fig. 2 shows that agricultural production activities have four basic stages, i.e., seeding preparation, cropland cultivation, crop growth and harvesting. Carbon emissions occur largely in the agriculture materials inputs (e.g. fertilizers, pesticides), farmland operations (tillage methods). In contrast, the processes of carbon sequestration occur through the growth of crops, straws and roots.

In summary, the annual carbon emissions and sequestration of household production activities are illustrated in Eq. 7, the index a represent the type of agriculture activities:

(7) $$\begin{array}{ccc} \text{Carbon}\;\text{flu}x_{\text{ia}} & = & \sum \left(\text{Emission}\;\text{facto}r_{a}*\;\text{Material}\;\text{inpu}t_{\text{ia}}\right)+\sum \left(\text{Emission}\;\text{facto}r_{t}*\text{Field}\;\text{Siz}e_{\text{ia}}\right)\\ & & +\;\sum \text{Biomas}s_{v}\;*0.475 \end{array}$$

– $\text{Carbon}\;\text{flu}x_{\text{ia}}$ represents the sum of carbon emission and carbon sequestration generated from agriculture activities of the household i.

In addition, at the final harvesting stage, the produced grains might be consumed or stored by the household or sold to others. The storage processes are assumed with no carbon emissions, while the sold grains are potentially consumed in other regions and thus emit carbon. Since the sold parts are no longer relevant to the surveyed households or the studied area, they are not considered in this research.

### 3.4 Carbon footprint of afforestation and economic tree plantation

Afforestation and economic tree plantation is another major carbon sequestration section in the mountain areas of west China [19]. In the recent decade, citrus planting has been rapidly developed in the TGR areas as local governments promoted economic development through the citrus industry. With this context, we calculate the total carbon sequestration of citrus per household according to equation 6:

(8) $$\;\text{Carbon}\;\text{Sequestratio}n_{\text{iaf}}=\text{Field}\;\text{Siz}e_{\text{iaf}}*\text{Carbon}\;\text{stoc}k_{\text{citrus}}$$

– $\text{Carbon}\;\text{Sequestratio}n_{\text{iaf}}$ represents the carbon sequestration generated from afforestation and economic tree plantation activities of the household i.

As reported in the study of [20], the carbon sequestration of citrus trees is about 222.80 $\text{tC}/hm^{2}$, which includes biomass and soil organic matter (SOM). Among the two, biomass sequestration only accounts for 10.14% of the total carbon sequestration, with a value of 22.58 $\text{tC}/hm^{2}$ [20], and SOM accounts for 200.21 $\text{tC}/hm^{2}$ (89.86% of total carbon sequestration). The household consumption of fertilizers, pesticides and herbicides are recorded as single values without specific application purposes, thus its carbon emissions are calculated in the agriculture category and not repeated in the afforestation activities.

### 3.5 Carbon footprint of livestock raising

**Fig. 3:**
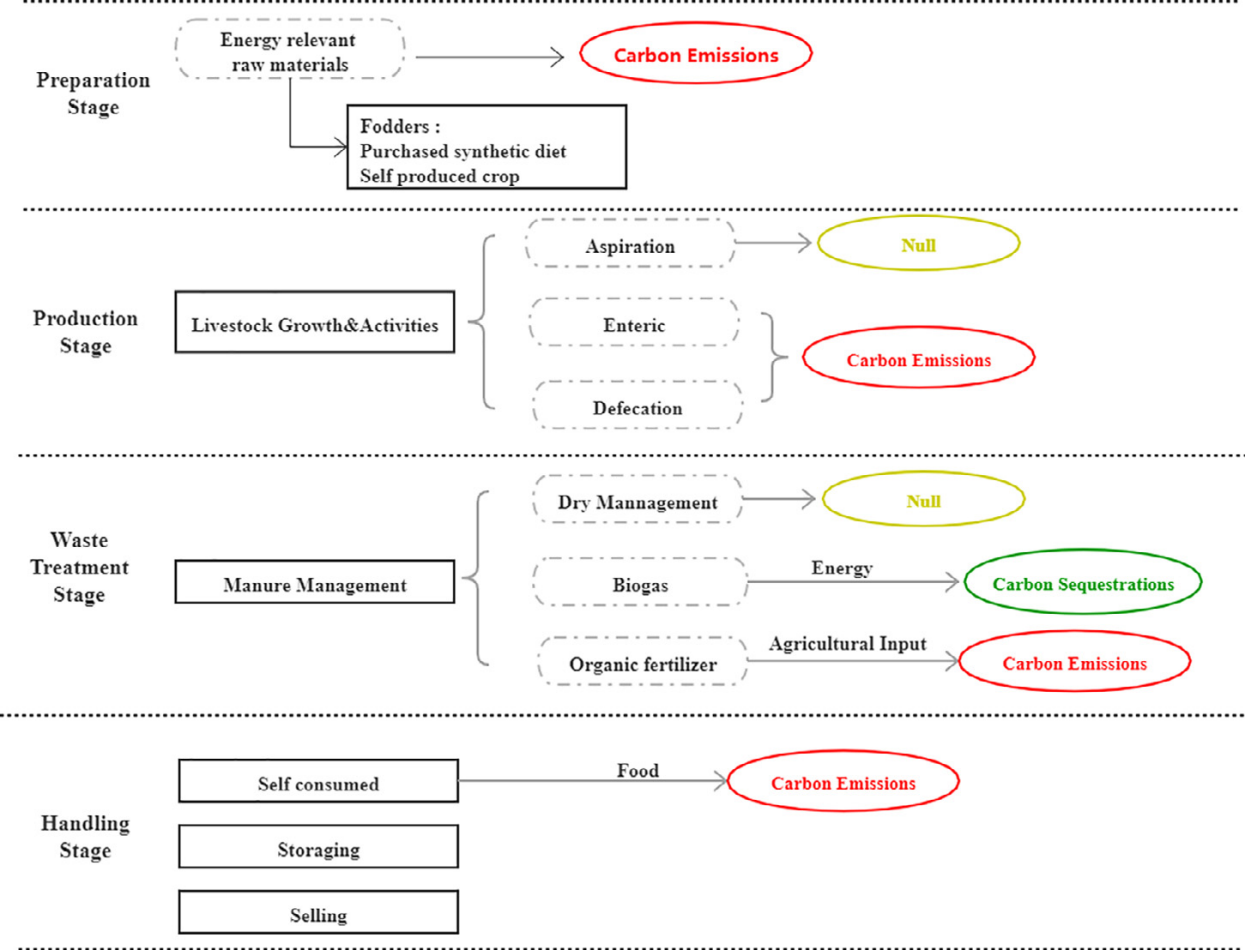
Life cycle process of livestock rising activities.

The carbon footprint of livestock raising by households was assessed with consideration of its four stages (Fig. 3): fodder preparation, livestock growth, manure management and livestock trading. Carbon emissions occur during fodder consumption, livestock rumination and excretion activities, and are mainly from $CH_{4}$ emissions that are largely generated by livestock enteric processes and excretion.

The annual carbon footprint of livestock raising are calculated according to equation 7:

(9) $$\text{Carbon}\;\text{emissio}n_{\text{il}\;}\;=\sum \left(\text{Emission}\;\text{facto}r_{\text{if}}*\text{Fodde}r_{\text{if}}\right)+\sum \left(\text{Emission}\;\text{facto}r_{\text{il}}*\text{Numbe}r_{\text{il}}\right)$$

– $\text{Carbon}\;\text{emissio}n_{\text{il}}$ represents the carbon emission generated from raising livestock activities of the household i.

The final livestock trading stage is the same as the trade process of crops, which is thus neglected in the present study.

## 4 Validation of the method

The LCA framework developed in this study was applied to a case study in the Three Gorges Reservoir area in west China, which acts as a validation process. We utilized the household survey data collected from the case area to test the feasibility of the framework.

### 4.1 Input data

The input data was taken from a large-scale household survey in selected districts and villages at the TGR area in 2016 and 2017. The TGR area has a subtropical monsoon humid climate with an average annual temperature of approximately 18 °C. The annual rainfall is approximately 1000–1200 mm, which is abundant but temporally and spatially uneven [21]. The main vegetation groups are coniferous forest, broad-leaved forest, bamboo forest and shrub. The terrain of the TGR area is complex and varies, with mountains accounting for over 74%, hills accounting for 21.7%, and valleys and small plains accounting for less than 4.3% [22]. Three types of villages were selected: two in the hilly area, two in the relatively flat area in the midst of valleys, and two near riverbanks with a significant number of migrants. In total, 422 valid questionnaires were collected from the six villages.

A survey household is defined as a group of people living together in one house that is considered as their primary home. Out-migrant workers who do not have another independent home are considered members of the household. Accordingly, the annual household income is the sum of incomes from all household members, including their employment, private enterprise, agricultural income, pension and government subsidies. The survey relies on the accounting of households’ daily spending and incomes. To ensure the data accuracy, the survey thus targeted to talk with the head of a household, who is in charge of the household economic management. In total, 422 valid questionnaires were collected from the TGR area.

**Fig. 4:**
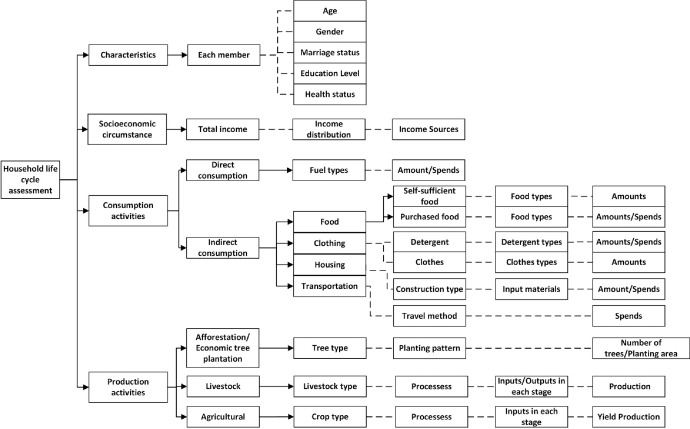
Survey data contents.

The survey data covers 100 questions of four major aspects (Fig. 4) regarding:

1) Basic characteristics of the household, including all members’ age, gender, marriage status, education level, profession, health status, etc.
2) Socioeconomic characteristics, including the main sources and amounts of household income as well as the investments and benefits of their economic activities.
3) Household consumption, including mainly the direct and indirect energy consumption associated with transportation, housing, clothing, food consumption, and other daily living expenses. The survey relies on people's memory and awareness. Thus the survey targeted to talk with the head of a household to obtain annual household data.
4) Household production activities, such as crop production, livestock raising and forestry. The detail in terms of all the input materials, land size, crop type, tillage method, irrigation method and all yield production are required in the survey data.

### 4.2 Data application

The detailed survey data could apply to the comprehensive life cycle assessment framework to evaluate a household's environmental influences (carbon footprint). Without the comprehensive life cycle assessment framework, the survey data might be failed to present the household carbon footprint. First, the detailed survey would record the same data in different categories, such as the gasoline consumption data might be re-collected in the transportation category. Second, the output products through one production category might be re-consumed by other consumption or production activities. For example, crop production output might be consumed in the food consumption or livestock feeding processes. Third, the survey data could be recorded by different units in terms of amount or spending which need to be converted to a uniform form.

The household life cycle assessment framework sets up a scope for the carbon footprint calculation with a clear boundary of each consumption and production activities. The innovation of presenting the clear corresponding life-cycle for each household activities firstly improves the explicitness of consumption and production in each category; secondly avoids the duplicate carbon footprint calculation of same consumption from different category and offset the value both existing in the consumption and production categories. For example, the agricultural production activity is exhibited by the whole processes from the preparation stage to the handling stage, and its relevant carbon footprint LCA will be the LCA processes. Another very inspired improvement of this research is the inclusion of household afforestation activities, which rarely existed in former studies. The household LCA framework described in this article could be widely used for households in urban or rural areas because it covers both consumption and production activities.

Despite these clear advantages, the household LCA framework does not include household received service goods. Future research could further expand the household LCA framework by including the carbon footprint of consumed service categories, which might be mostly characterized by urban households, such as take-away food delivery, online shopping delivery, etc. Assessing the carbon footprint of these modern service activities would provide an insight into the household environmental influence differences between urban and rural households.

### 4.3 Next research steps

As shown in the co-submission paper, the improved LCA framework produced valuable information on the carbon footprint of rural households in the mountainous west China. To reach a further enhancement of the study, next research steps are expected to:

• Include the carbon footprint of service products, which are expected to increase fast in the rural areas along with the general social-economic development.
• Apply the LCA framework in various regions with diverse rural livelihoods, e.g. in geographically plain areas in East China, and in developed countries in Europe.
• Compare the carbon footprint of rural households and urban households, and thus to enable an integrated assessment of carbon footprint in a rural–urban system.

## Declaration of Competing Interest

The authors declare that they have no known competing financial interests or personal relationships that could have appeared to influence the work reported in this paper.
